# A comparison of the effects of occupation-based interventions with and without responsibility feedback and conventional interventions on participation in people with idiopathic Parkinson’s disease: study protocol for a randomized controlled trial

**DOI:** 10.1186/s13063-023-07526-3

**Published:** 2023-09-26

**Authors:** Mahsa Meimandi, Akram Azad, Jafar Haj Ghani, Fatemeh HojabriFard, Philip von Rosen, Naeeme Haji Alizadeh, Ghorban Taghizadeh

**Affiliations:** 1 Rehabilitation Research Center, Department of Occupational Therapy, School of Rehabilitation Sciences, Iran University of Medical Sciences, Shahnazari Street, Mirdamad Boulevard, Tehran, Iran; 2 Department of Occupational Therapy, School of Rehabilitation, Shahid Beheshti University of Medical Sciences, Tehran, Iran; 3 Division of Physiotherapy, Department of Neurobiology, Care Sciences, and Society (NVS), Karolinska Institutet, Huddinge, Sweden; 4 Department of Neuroscience, School of Advanced Technology in Medicine, Iran University of Medical Sciences (IUMS), Tehran, Iran

**Keywords:** Occupations, Occupational therapy, Parkinson’s disease, Participation, Rehabilitation

## Abstract

**Background:**

Parkinson’s disease (PD) is a neurodegenerative disorder with debilitating motor and non-motor symptoms which affect participation in meaningful occupations. Occupation-based interventions can improve participation in people with PD. Evidence for incorporating structured and intensive occupational therapy by considering the concept of responsibility is lacking for this population. This trial will compare the effects of occupation-based interventions with and without responsibility feedback and conventional interventions on participation in people with idiopathic PD.

**Methods:**

A total of 45 people with PD, between 35 and 85 years old and Hoehn and Yahr stages between I to III, will be recruited from movement disorder centers for this three-armed study. Participants will be randomized into three groups (occupation-based interventions with responsibility feedback, occupation-based interventions without responsibility feedback, and conventional interventions). All participants will receive intervention for 24 sessions during a period of 12 weeks (2 sessions per week). The primary outcome measure will be participation satisfaction. Participation frequency and restriction, self-perceived performance, performance satisfaction, motivation, volition, sense of agency, responsibility, physical activity, community integration, activities of daily living (ADL), instrumental ADL, upper extremity function, balance, fatigue, and quality of life will be measured as secondary outcome measures. All outcomes will be measured at baseline, session 9, session 17, post-intervention (week 13), and follow-up (week 25).

**Discussion:**

This home-based high-intensity, structured, client-centered, and occupation-based intervention will be conducted by utilizing the concept of responsibility. This proposed trial may result in enhanced participation that would benefit other motor and non-motor symptoms in people living with PD. Findings from this proposed study are expected to expand the knowledge of clinicians and help them in evidence-based decision-making processes.

**Trial registration:**

Iranian Registry of Clinical Trials IRCT20140304016830N13. Registered on August 19, 2022

**Supplementary Information:**

The online version contains supplementary material available at 10.1186/s13063-023-07526-3.

## Background

Parkinson’s disease (PD) is recognized with debilitating motor and non-motor symptoms [1, 2]. Non-motor complications lead to the limitations in activities of daily living (ADL), restricted participation in meaningful occupations, and decreased quality of life [3–5].

Reduced basal ganglia output in PD plays a critical role in motivation [6, 7] and volition [8] brain circuits. The literature proposed that the relation between motivation and volition affects participation [9–11]. Participation in occupations includes motivational (i.e., pre-decisional and post-actional) and volitional (i.e., pre-actional and action) processes [10, 12]. Motivational and volitional levels are prerequisites for participation and adherence to therapeutic interventions [13]. Occupational therapy (OT) with a client-centered and occupation-based approach can address these issues [14, 15].

Based on the literature, a sense of responsibility affects motivation, volition, and ultimately participation [8, 16, 17]. People living with chronic diseases (e.g., PD) tend to lose their sense of responsibility towards their daily occupations and feel incapable of managing their lives [18, 19]. Hence, interventions based on this concept may be beneficial for increasing motivation, volition, and participation.

Over the past two decades, most intervention studies for this population were multidisciplinary (including OT) [20–24]. Only two recent large-scale studies were conducted with a client-centered approach [25, 26]. Sturkenboom et al. [25] compared the efficacy of home-based OT in Parkinson’s disease (OTiP) with usual care (no OT) over a 10-week program (average of 8 1-h sessions). Self-perceived performance and satisfaction in daily activities were improved but participation did not benefit from this intervention. Clarke et al. [26] delivered low-dose (i.e., median 4 1-h sessions in 8 weeks) individualized physiotherapy and OT in mild to moderate PD and concluded no immediate or medium clinically meaningful improvements in ADL or quality of life. They concluded that low-dose therapy in early PD stages is ineffective and suggested more structured and intensive programs. A recent systematic review [27] indicated that drawing rigorous conclusions on OT intervention approaches is currently impossible. Literature recommended that upper extremity and ADL functioning should be addressed simultaneously with high-intensity programs. Also, this study suggested that non-motor symptoms require further attention.

The concept of sense of responsibility was not considered in the aforementioned studies while delivering interventions. Sense of responsibility refers to the intentional binding mechanism that connects our intentions to the corresponding outcomes. We attribute our own activities to ourselves in this process; hence, we feel a sense of control and accountability [28, 29]. Sense of responsibility is intertwined with agency (i.e., controlling doing of occupations and their consequences) and its neural correlates. These neural correlates (e.g., angular gyrus, insula, supplementary motor area) play a critical role in agency and responsibility [30, 31]. Sense of responsibility and agency are crucial while executing goal-directed occupations [32–34] and may change health-related behaviors, such as non-motor symptoms [35, 36]. Moreover, feedback (knowledge of result or knowledge of performance) may cause behavioral and neurological changes and hence promote participation [37]. This study aims to conduct a trial to compare the efficacy of occupation-based interventions on participation in people living with idiopathic PD in three groups: (1) occupation-based interventions with responsibility feedback, (2) occupation-based interventions without responsibility feedback, and (3) conventional interventions.

## Methods

### Trial design

**Fig. 1:**
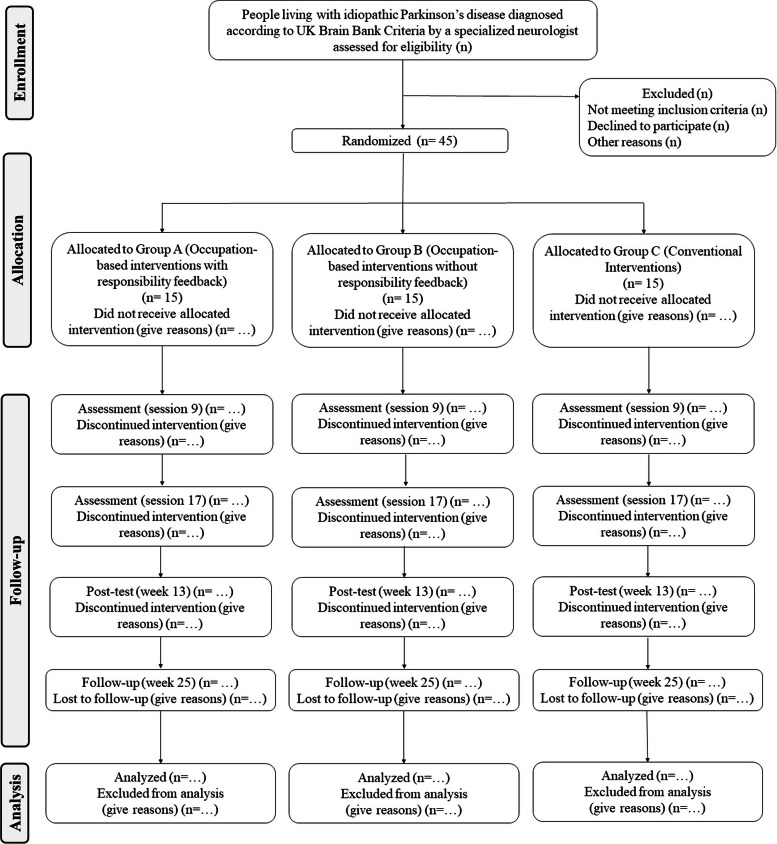
Flow diagram illustrating trial design for people with idiopathic Parkinson’s disease

A parallel, three-armed, double-blinded (assessor and patients) randomized controlled trial will be conducted. Individuals will be assigned to each group in a ratio of 1:1:1 (group A: occupation-based interventions with responsibility feedback; group B: occupation-based interventions without responsibility feedback; group C: conventional interventions). The CONSORT diagram [38] will present the trial design (i.e., enrollment, allocation, and assessments) in brief (Fig. 1). This clinical trial follows the recommendations for Interventional Trials (SPIRIT) [39] statements (Additional file 1).

### Participants and recruitment procedures

Community-dwelling individuals with Parkinson’s will be recruited from movement disorder centers and rehabilitation clinics in Tehran, Iran. Advertisements in Iran’s Parkinson Association will be done. Individuals who meet the inclusion criteria (see section below) will receive consent form and information regarding study aims and procedures.

Due to COVID-19 pandemic precautions, patients will only come to the OT clinic for evaluations. All sessions will be free for participants, and travel costs for evaluation will be paid by the research team. Both assessments and interventions will be conducted in the “ON” (1 h after levodopa intake) medication state. Levodopa equivalent dose (LED) will be calculated for individuals in each assessment session using Tomlinson et al. formula [40].

### Data collection

The assessor (an occupational therapist with 6 years of experience in neurological rehabilitation) will conduct initial screening and outcome measure evaluations at baseline, session 9, session 17, post-intervention (week 13), and follow-up (week 25) (Table 1). Since this is a low-risk study, independent data monitoring and auditing will not be taken into consideration. Written feedback about the results of the evaluations will be provided to all participants in order to promote retention. A recent systematic review suggested that people with PD will benefit from moderate to high doses of intervention (≥ 8 sessions and ≥ 10 weeks) [5]. We will intend to discover the proper dose of intervention by multiple assessments during the treatment process. Treatment sessions will be started 1 or 2 days after the initial screening in participant’s homes for 12 weeks (two times a week) by three experienced occupational therapists (average 5 years of experience). The occupational therapists will be trained for 2 days regarding the study procedures and intervention protocols. Therapists will be allowed to contact the main researcher to share any issues that arisen in the intervention process. Home-based therapy with a focus on meaningful occupations appears to be the most plausible beneficial intervention [27]. Moreover, the environment is more familiar for the patient, and near-ones find it more convenient [41].

**Table 1:**
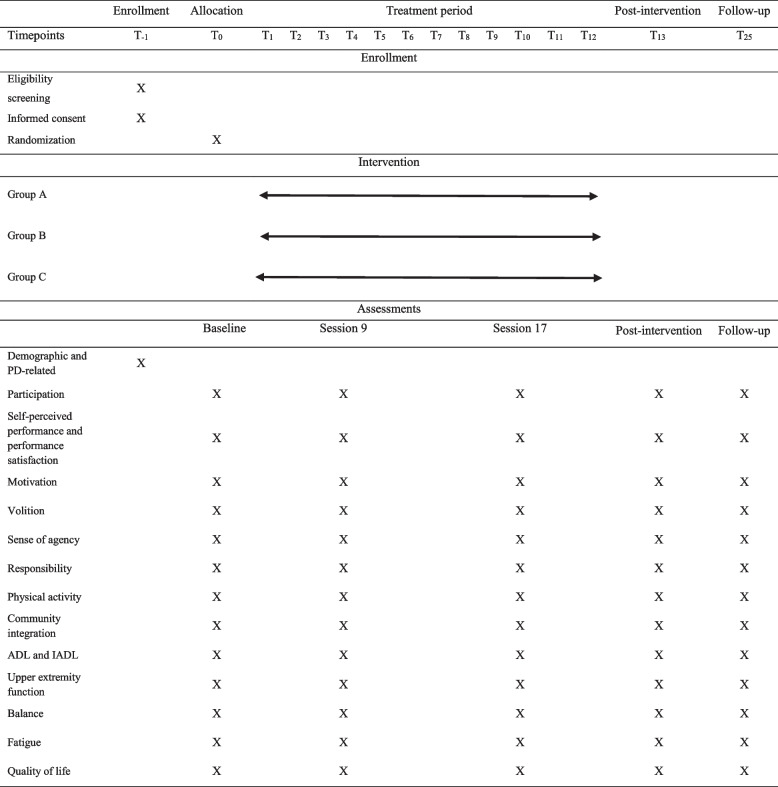
Schedule for treatment and outcome measures

Group A: occupation-based interventions with responsibility feedback. Group B: occupation-based interventions without responsibility feedback. Group C: conventional interventions

*PD* Parkinson’s disease, *ADL* Activities of daily living, *IADL* Instrumental ADL

### Inclusion and exclusion criteria

The inclusion criteria will be (a) diagnosis of idiopathic PD based on the UK Brain Bank criteria [42], (b) absence of impulsive-compulsive disorder based on interviews by the neurologist and published criteria [43–45], (c) Hoehn and Yahr stages of I to III [46], (d) aged between 35 and 85, (e) acceptable cognitive function (a score of > 24 on Montreal Cognitive Assessment [47]), and (e) able to read and write in Persian for completing self-report questionnaires. The exclusion criteria will be (a) atypical parkinsonian syndromes [48]; (b) other existing neurological disorders, such as stroke; and (c) juvenile PD.

### Randomization and blinding

After obtaining consent and baseline data, an independent researcher who will not be involved in assessments will allocate the participants to each group. Participants will be randomly assigned to each group using a sequence generated by http://www.randomizer.org. For determining concealment, unique codes will be written on envelopes which contain group specifications. The assessor will be blind to the allocation of participants in groups and will enter data in the OT clinic. We do not anticipate any requirement for unblinding but if required, the trial steering committee (i.e., chief investigators and clinical project manager) will have access to the group allocations and any unblinding will be reported. Assessments will be conducted before allocation. Moreover, patients will not know which intervention they are receiving.

### Sample size calculation

The sample size was estimated using the G-Power software with Cohen’s *f* standardized effect size of 0.25, correlation among repeated measures of 0.25, power of 80%, and first type error of 5% based on the primary outcome measure (i.e., Utrecht Scale for Evaluation of Rehabilitation-Participation Satisfaction) [49]. The total sample size was calculated as 39 subjects which increased to 45 subjects by assuming a 15% dropout rate. In order to assure the sufficiency of the sample size, power calculation will be performed again at the end of the trial.

### Interventions

First, the importance of therapy in people with PD will be explained to participants. In each group, treatment sessions will be delivered two times a week (Sunday/Tuesday, Saturday/Wednesday, or Monday/Thursday) for 12 weeks. Each session will be approximately 60 to 90 min. Treatment sessions will be performed in the morning or evening based on the participant’s convenience and medication regime (i.e., being in “ON” state while receiving treatment). Rest will be provided for each patient as required. Participants will be instructed not to receive any other types of rehabilitation intervention during the treatment sessions. Moreover, the intervention will be discontinued when participants express their desire to terminate the intervention.

### Occupation-based interventions with responsibility feedback (group A)

This group will receive occupation-based interventions with responsibility feedbacks. These occupations and feedback statements were derived from international Delphi studies with renowned experts and national Delphi studies with people living with PD [28, 50]. Responsibility towards oneself, family members, friends, and other people in society was considered in these studies. Occupations deemed as having a very high to moderate inherent sense of responsibility based on consensus among experts and patients were chosen for designing the intervention. The derived occupations were ADL, instrumental ADL (IADL), health management, and leisure, such as maintaining personal hygiene, meal preparation and cleanup, and medication management. These occupations were approximately in common with Clarke et al.’s study [26].

The aforementioned occupations will be graded based on Gentile’s taxonomy [51]. Two general features (i.e., environmental context and function of the action) are considered in this taxonomy. Environmental context involves two characteristics with or without intertrial variability: (1) stationary regulatory conditions and (2) in-motion regulatory conditions. The regulatory conditions include surface (soft or hard/ rough or smooth/flat or inclined), space (wide or narrow), and barrier features. The function of the action encompasses body stability and body transport with or without object manipulation. Discrepancies regarding the difficulty levels of the Gentile taxonomy for each occupation were discussed and resolved in a panel of 11 experts including occupational therapists and physical therapists with at least 5 years of experience in PD rehabilitation (Table 2).

**Table 2:** Gentile taxonomy and an example of occupation grading using this taxonomy

Environmental context	Action function				
	Body stability		Body transport		
	No object manipulation	Object manipulation	No object manipulation		Object manipulation
Stationary regulatory conditions and no intertrial variability	1A (body stability no object)	1B (body stability object)	1C (body transport no object)		1D (body transport object)
Stationary regulatory conditions and intertrial variability	2A (body stability no object)	2B (body stability object)	2C (body transport no object)		2D (body transport object)
In-motion regulatory conditions and no intertrial variability	3A (body stability no object)	3B (body stability object)	3C (body transport no object)		3D (body transport object)
In-motion regulatory conditions and no intertrial variability	4A (body stability no object)	4B (body stability object)	4C (body transport no object)		4D (body transport object)
*An example of occupation grading using Gentile taxonomy*					
Occupation	Occupation components	Difficulty components		Difficulty level	
Dressing	1-Grabbing the T-shirt 2-Folding the T-shirt 3-Raising the T-shirt for wearing 4-Putting the arms in sleeves 5-Pulling the T-shirt down 6-Grabbing the T-shirt 7-Pulling the T-shirt up 8-Taking off the T-shirt 9-Putting the T-shirt in a proper place	-Position of the individual in relation to the T-shirt or vice versa -Pace -T-shirt status or position -Environment (e.g., lighting, temperature) -Dual task (e.g., simultaneous cognitive task) -Others (e.g., shape, material, weight)		1B: sitting or standing position, pre-defined procedure within a fixed environment 2B: sitting or standing position, various performance methods within a variable environment 3B: walking, pre-defined procedure within a fixed environment 4B: walking, various performance methods within a variable environment	
*Examples of responsibility feedback statements*					
You will feel better and more satisfied with life by doing activities/occupations that you have recently gained the ability to do.					
By doing this occupation, you reduce the burden on your family members.					
You are the main interested in your improvement.					
Doing this occupation gives you a sense of empowerment.					
Doing this occupation helps you to work towards becoming independent again.					
Not doing this occupation will be disadvantageous to your family members.					

The functional level of each patient will be determined using the Gentile taxonomy. The intervention will be delivered to patients at any level of the taxonomy where they experience challenges. Participants will receive occupations from simple (i.e., stationary condition with no intertrial variability and object manipulation) to hard (i.e., in-motion conditions with intertrial variability and object manipulation) levels based on their priorities identified via Canadian Occupational Performance Measure. All patients will be actively participating in at least three to four occupations. Therefore, all participants will receive the same amount of intervention. The therapists will keep a record of the extent of participation in each occupation using a visual analog scale during each session.

Moreover, feedback statements that have the potential to create a sense of responsibility (i.e., derived from international and national Delphi studies with renowned experts and patients) in patients will be provided to participants as knowledge of performance or knowledge of the result. These feedback statements will be positive or negative statements in relation to the individual or his/her family for prevention/symptom management and function improvement. These feedback statements (i.e., based on type, target, and purpose) will be presented to the patients throughout the intervention sessions. An expert panel was held to sort the feedback statements so that patients receive all forms of feedback in each session. Some examples of responsibility feedback are provided in Table 2.

This group will receive conventional interventions (see below) for 15 min in each session. They will receive occupation-based interventions with responsibility feedback for the remaining 45 to 75 min.

### Occupation-based interventions without responsibility feedback (group B)

Participants in this group will receive occupation-based interventions (described above). Responsibility feedbacks will not be provided to this group. This group will also receive 15-min conventional interventions and 45- to 75-min occupation-based interventions.

### Conventional interventions (group C)

All the participants who are assigned to this group will only receive traditional treatment (60 to 90 min), including passive mobilization, lower extremity strengthening, stretching, motor coordination (upper and lower extremity), balance and walking training, manipulation exercises, breathing, relaxation, and postural exercises [52, 53]. Depending on the condition of each patient, they will receive these interventions. The duration and number of each therapeutic intervention will be recorded in each session.

### Measurements

Demographic and PD-related information including age, gender, education, family status, marital status, job status, assistive device use, fall history, years since diagnosis, and medications will be collected from participants. Outcome measures will be assessed by a blind certified occupational therapist at baseline, session 9, session 17, post-intervention, and follow-up.

### Primary outcome measure

The primary outcome of this trial will be participation satisfaction assessed with the Utrecht Scale for Evaluation of Rehabilitation-Participation Satisfaction Scale (USER-P). The satisfaction scale consists of 10 items including vocational activities, leisure activities, and social relationships. Each item will be rated on a 5-point Likert scale. The summation of scores will be converted to a 0–100 scale, with higher scores indicating greater participation satisfaction [54]. The Persian version of the USER scale has shown appropriate validity and reliability [55].

### Secondary outcome measures

Secondary outcomes will include participation frequency and restriction, motivation, and volition as well as sense of agency, responsibility, physical activity, community integration, ADL and IADL, upper extremity function, balance, fatigue, and quality of life.

Several psychometric scales will be employed for these secondary outcome measures, as follows:

- Participation frequency and restriction

*Frequency and restriction scales of USER-P* will be regarded for measuring this outcome. The frequency scale consists of two parts. Part A measures the time (0 = not at all; 5 = 36 h or more) spent on paid work, unpaid work, volunteer work, and housekeeping. Part B measures the frequency (0 = not at all; 5 = 19 times or more) of social and leisure activities. The restriction scale evaluates restrictions experienced while participating in daily activities. Items will be scored as not applicable (NA), not possible (1), with assistance (2), with difficulty (3), and without difficulty (4). The sum scores in these two scales will be converted into a 0–100 scale (54).

- Self-perceived performance and performance satisfaction

*Canadian Occupational Performance Measure* will be used to evaluate the self-perceived occupational performance and satisfaction with a semi-structured interview in areas of self-care, productivity, and leisure [56].

- Motivation

*Social Motivation Questionnaire* will be used to measure the information-seeking (4 items) and emotion-regulatory behavior (4 items) motivation for social participation. Each item will be graded between 1 (very disagree) and 7 (very agree). Higher scores will denote stronger information-seeking and emotion-regulatory behavior motivation [57].

- Volition

*Volitional Questionnaire* is a MOHO tool which will evaluate volitional development via observing individuals while performing occupations with 14 items. Items will be rated as follows: 1 = passive, 2 = hesitant, 3 = involved, and 4 = spontaneous. Higher scores will represent a higher level of volition [58].

- Sense of agency

*Intentional Binding Task* measures the implicit sense of agency. Subjects will be asked to judge the time of actions (i.e., key press) and effects (i.e., tones) via a clock hand rotating around a clock face on a computer screen. This task contains two baseline (tone, action) and two operant (tone, action) conditions [59].

- Responsibility

*Allocation of Treatment Responsibility Scale* will evaluate the responsibility during medication-related tasks for caregivers and patients with two parallel forms. Each item is rated from 1 (none of the time) to 4 (all the time). The total score will be the summation of items, with higher scores reflecting increased perceived responsibility [60].

- Physical activity

*Phone Frequency, Intensity, Time, and Type (Phone-FITT) Questionnaire* will measure the physical activity participation in household (6 items) and recreational (11 items) activities. The frequency and duration of each activity will be asked. The duration is rated on a 4-point scale (0 = 0 min; 1 = 1–15 min; 2 = 16–30 min; 3 = 31–60 min; 4 = 1 h and more). Higher scores will indicate greater levels of physical activity participation [61].

- Community integration

*Community Integration Questionnaire-Revised* will evaluate the individual’s integration into home and community with 18 items. Most items will be rated on a scale of 0 to 2, with 2 representing greater integration. Subscale scores will be calculated as follows: home integration (0–12), social integration (0–10), productivity (0–7), and electronic social networking (0–6). Higher scores will reflect a higher degree of community integration [62].

- ADL and IADL

*Nottingham Extended ADL Scale* will be used to assess the level of independence in mobility, kitchen, domestic, and leisure activities. Each item will be scored with 4 (no (0 points), with help (1 point), on my own with difficulty (2 points), and on my own (3 points)) response options. A higher total score will indicate greater independence [63].

- Upper extremity function

*9-Hole Peg Test* will be used to evaluate hand dexterity. The participant will be asked to pick pegs one by one from the container and place them into the holes on the board as quickly as possible. Participants must then remove the pegs one by one and replace them back into the container. The time taken to complete this procedure will be recorded for each hand [64].

- Balance

*Dynamic Gait Index* will evaluate the patient’s ability to maintain balance while walking with different task demands in 8 dynamic conditions. Items will be rated on a 4-point Likert scale. The total score will range between 0 and 24 points, with higher scores denoting better performance [65].

- Fatigue

*Fatigue Severity Scale* will be used to measure the effect of fatigue on daily functioning. This scale consists of 9 items, rated from 1 (completely disagree) to 7 (completely agree). Higher scores will indicate severe fatigue [66].

- Quality of life

The *Parkinson’s Disease Questionnaire (PDQ-39)* will be used to evaluate health-related quality of life across dimensions of daily living. Items will be rated on a 5-point Likert scale (0 = never; 4 = always). Each domain’s score will be calculated by the sum of scores of items in the domain divided by the maximum possible score of the domain, multiplied by 100 [67].

### Data analysis

Data will be entered and analyzed using SPSS version 13. Each participant will have a registration number. All printed questionnaires and datasets will be stored in a secure locker and computer, respectively. Missing data will be handled by random coefficient analysis [68]. Intention-to-treat analysis will be performed for dropout participants during treatment sessions or follow-up assessments. Dropout participants will not be excluded from the sample size and data analysis. There will be no planned interim analyses and no planned stopping rules for this trial. The mean (standard deviation) or median (inter-quartile range) will be calculated for quantitative variables. For categorical variables, frequency (percentages) will be used. To check the normality of data, Shapiro-Wilk, graphical methods, and numerical indices will be used.

Quantitative demographic variables between the three groups will be compared using one-way ANOVA or Kruskal-Wallis tests. Chi-squared will be used for comparing the categorical variables between the groups. If there will be no significant difference between various outcome measures in the three groups at baseline, outcome measures will be analyzed using a 3 × 5 repeated measure analysis of variance with group (occupation-based interventions with responsibility feedback; occupation-based interventions without responsibility feedback; conventional) as between-subject factor and time (baseline, session 9, session 17, post-intervention, follow-up) as within-subject factor. A Tukey post hoc test will be performed for multiple comparisons. However, if a significant difference will be obtained for outcome measures in the three groups at baseline, a separate regression analysis will be done by considering group and baseline as covariates at each assessment point (i.e., session 9, session 17, post-intervention (week 13), follow-up (week 25)). The differences will be calculated via Kruskal-Wallis. All analyses will be considered significant with a 2-tailed *P* < 0.05.

## Discussion

This trial will be innovative with a novel aspect that could pave the way for future studies. Although previous studies [25, 26] have investigated the effect of individualized OT, some methodological issues (i.e., intensity and structure of intervention) impede drawing rigorous conclusions. Moreover, a recent systematic review indicated that these individualized OT interventions provide a low to moderate strength of evidence for improving IADL performance and participation [5].

In response to these issues, this high-intensity, structured, client-centered, and occupation-based trial will be conducted by utilizing the concept of responsibility. This intervention will be delivered with a home-based approach. Therefore, future studies can compare the cost-effectiveness of this intervention in relation to interventions implemented in outpatient settings. Findings from this proposed study are expected to expand the knowledge of clinicians and help them in evidence-based decision-making processes.

This study will have some limitations. First, intervention delivery may be disrupted due to weather and therapist or patient sickness. Available therapists within the team will be incorporated to cover sickness leaves. Second, the results can only be generalized to people with PD who receive home-based interventions. Third, patients may underestimate or over-report the outcomes of the trial due to the use of self-report measures for evaluation. Fourth, patients may experience “on/off” fluctuations during assessment sessions.

In conclusion, the results of this proposed trial may result in enhanced participation (i.e., the ultimate goal for both patients and therapists) that would benefit other motor and non-motor symptoms in people living with PD.

## Trial status

The conception of the occupations and responsibility feedback statements are finished. Recruitment started in September 2022. It is expected that assessments for the last included patients will be completed by June 2023. If any modifications will be needed in the protocol (including study design or procedures), it will be agreed upon by the Iranian Registry of Clinical Trials. The current protocol (Code: IRCT20140304016830N13) is version 1.0, dated 19 August 2022.

## Availability of data and materials

The datasets used and analyzed during the study will be available upon reasonable request from the corresponding author. Questionnaires and evaluation forms for each participant will be stored in a secure locker. A code will be assigned to each participant’s folder. Double data entry and range checks for data values will also be done to promote data quality.

### Supplementary Information

**Additional file 1.** 

## Abbreviations

PD: Parkinson’s disease
ADL: Activities of daily living
OT: Occupational therapy
MOHO: *Model of Human Occupation*
OTiP: Occupational Therapy in Parkinson’s Disease
LED: Levodopa equivalent dose
IADL: Instrumental activities of daily living
USER-P: Utrecht Scale for Evaluation of Rehabilitation-Participation
Phone-FITT: Phone Frequency, Intensity, Time, and Type
PDQ-39: Parkinson’s Disease Questionnaire

## Declarations

Ethical approval and consent to participate

This study obtained ethical approval (IR.IUMS.REC.1400.753) on 21 November 2021 from the Ethics Committee of the Iran University of Medical Sciences. The first author (MM) will obtain informed consent from all individuals prior to any procedures. Study participants will be able to withdraw from participating at any time. Participants will be asked on the consent form if they approve of the usage of their data in the event that they choose to withdraw from the study. Participants will also be requested for their consent to allow the research team to share pertinent data with individuals from the university. We will not collect biological specimens in this trial.

The trial results will be published in scientific journals and presented at neurology or occupational therapy conferences. Participants will be able to receive information about the trial’s findings.

Patient/Public Involvement:

Since there has been a paradigm shift towards the active involvement of patients in research design in recent years, we have included patients’ opinions regarding the level of responsibility in occupations and determining the responsibility feedback statements and occupational therapy priorities.

Adverse event reporting and harms:

No physical or psychological harm was anticipated in this trial, although the Iran University of Medical Sciences Ethics committee will be informed of any potential issues, whether physical or mental, that emerge as a result of the treatment. As per the Ethics Committee’s rules, the study team will give precedence to unpaid evaluation and treatment. Given the predicted low risk of harm from a participant’s involvement in this research, there are no provisions for post-trial care.
